# Congenital digital flexural deformity (knuckling): Epidemiology, the association of trace elements and surgical treatment in neonatal bovine calves

**DOI:** 10.5455/javar.2023.j656

**Published:** 2023-03-31

**Authors:** Shrabony Chakraborty, Mst. Antora Akter, Md. Sabuj Rahman, Nelema Yesmin, Nasrin Sultana Juyena, Md. Mahmudul Alam

**Affiliations:** Department of Surgery and Obstetrics, Bangladesh Agricultural University, Mymensingh, Bangladesh

**Keywords:** Calves, congenital defect, flexural deformity, tendon trantsecion, tendon elongation, Z-tenotomy

## Abstract

**Objectives:**

This study aimed to investigate the prevalence and pattern of congenital flexural deformity (knuckling), to identify the association between trace elements and vitamins with the deformity, and to apply different surgical techniques for correcting this congenital malformation in newborn bovine calves.

**Materials and Methods:**

The study was implemented on 17 newborn calves with carpal (knee) and fetlock (foot) knuckling presented to the Veterinary Teaching Hospital of Bangladesh Agricultural University, Mymensingh, from January to December 2020. The serum biochemical alterations and clinical outcomes were assessed on days 0 and 21 following surgery. Two surgical methods: tendon transection and tendon elongation by Z-tenotomy, were performed for surgical restoration.

**Results:**

We found that knuckling comprised 12% of the total congenitally malformed calves. The male calves had a higher prevalence (52%, *n =* 9) and the same in the winter season (65%, *n =* 11). The majority of the knuckling was bilateral types (88%, *n =* 15), involving the carpal joint (82%, *n =* 14) and moderately angulated (59%, *n =* 10). The serum level of magnesium, iron, vitamin D, and zinc were significantly (*p *< 0.05) changed from the pre-surgical stage to the stage of non-lameness after surgery. The disorder was surgically treated by tendon transection or tendon elongation procedure and had a good prognosis.

**Conclusion:**

The current study concluded that the development of knuckling in calves might be related to a deficiency/excess of specific minerals and vitamins and that it can be effectively corrected by surgical intervention; however, early diagnosis and the use of proper surgical techniques are crucial for improving the prognosis.

## Introduction

The inability to acquire or maintain natural limb extension may result in the development of flexural limb deformities termed knuckling. This is the most common musculoskeletal anomaly in newborn calves [1]. Because flexural malformations can involve more than one structure, it is more realistic to address them in terms of the joints involved rather than the tendons and ligaments [2]. The most prevalent form of congenital limb deformity in cattle is the metacarpophalangeal flexural deformities (MPFD) of the forelimbs [3,1], but the carpal and coffin joints are also vulnerable [4]. Multiple joints may be involved in severe congenital cases, but most often, just one joint is affected [5]. These limb deformities can be classified as mild (self-correctable), moderate (correctable with therapy), and severe (rarely correctable). A calf with mild deformities could walk on the tip of its hoof [6], but its heels would not touch the ground. Moderately deformed animals could stand on the tip of the hoof but walk on the back of their ankles [7], and calves with severe deformity may stand or walk on the dorsal side of the foot, fetlock-foot junction, or the carpal joint [6]. Two major flexural abnormalities include extreme carpal angulation and arthrogryposis, characterized by neck bending and deformed carpal or tarsal joints. The knuckling is caused by inherited factors, improper uterine postures, insufficient nutrition, and shortages or excesses of vitamins and minerals [8,9]. The diagnosis can be determined with ease by observing the flexed limb during an inspection. Still, radiography may be helpful if there is cause to suspect both a joint lesion and a tendinous lesion [10,11].

The congenital malformation is one of the leading reasons behind the culling of animals, where knuckling contributes a significant role [6]. In the end, this has an important economic consequence. Animals with flexural abnormalities may be incapable of nursing; hence the inability to gain passive immunity transfer may be a complication. When there is a flexural deformity, an element of pain is involved. This suffering can lead to a worsening of the deformity and/or an overload and subsequent distortion of the contralateral limb [12]. This is one of the reasons why, in most cases, treatment should be initiated immediately upon diagnosis. If left untreated, the disease progresses to chronicity, skin ulcers on the fetlock’s dorsum, and ultimately septic arthritis [7]. Thus, immediate surgery is the best course of action to preserve the animal’s life and the farmer’s finances. 

Based on the facts mentioned above, the present study aimed to determine the epidemiology of knuckling, to assess certain minerals and vitamins for analyzing the etiology of the diseases, and to evaluate the surgical reconstruction of the superficial and deep digital flexor tendon (DDFT) by transection and elongation methods. 

## Materials and Methods

### Ethical approval

The animal work has been performed following the guideline of the Animal Welfare, Ethics, and Experimentation Committee [(AWEEC, permission number: AWEEC/BAU/2021(47)].

### Experimental animals 

The animals of this clinical study included 17 calves of different breeds, ages, and genders, which were brought to the Veterinary Teaching Hospital (VTH) of Bangladesh Agricultural University (BAU), Mymensingh, from January to December 2020 with the complaint of standing difficulty, unable to walk, and clinically visible of bending of knee and fetlock. 

### Clinical examination

**Figure 1.**
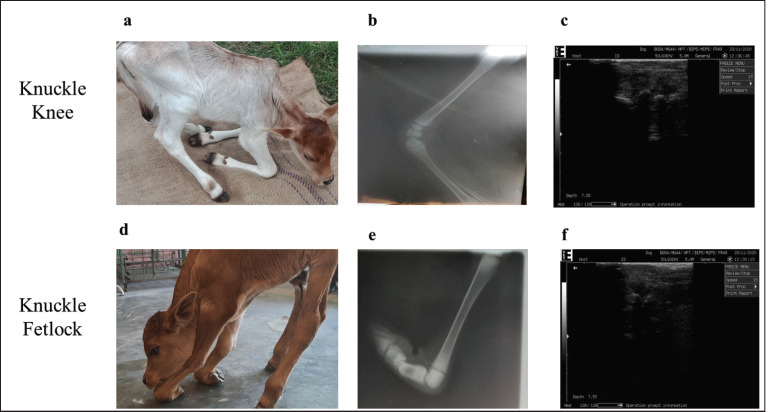
Preoperative clinical, radiographic and ultrasonographic examination in calves a) clinical appearance, b) radiographic examination and c) ultrasonographic examination in knuckle knee. Similarly, d) clinical appearance, e) radiographic examination and f) ultrasonographic examination of calves with knuckling of the fetlock.

Clinical examinations manifested that one or both the forelimbs were malformed, with the knee and/or fetlock flexed and that the patient could not maintain a flat limb (Fig. 1a and d). The joints’ distance and/or close inspection and passive flexure movements were used to detect congenital flexural deformity in the calves. Joints only partially or never reached the usual extension was diagnosed as congenital flexural deformity. The physiological parameters of temperature, heart rate, and respiration rate were recorded. 

### Radiographic and ultrasonographic examination

After initial physical and clinical assessment, preoperative radiography was performed on lateromedial positions to examine the angulations and joint deformities (Fig. 1b and e). The ultrasonographic examination was served with a 5 MHz linear probe to check the soundness of superficial and DDFT and associated structures (Fig. 1c and f).

### Determination of the knuckling angle

The angle of the knuckling was measured following the procedure described by Sirin et al. [13] with minor modifications. Briefly, the calves were confined without any prior medication in lateral recumbency. The affected limb was placed on the upside and extended faintly, and the angle was copied on a pain paper. A protractor was used to measure the angle made by an imaginary central longitudinal line on the lateral aspect of the metacarpal or carpal bone and the equivalent central line of the foot drawn on paper. After that, the opposing pairs of calves were confined so that the knuckle angle could be determined.

### Clinical grading of knuckling

The degree of flexural deformity in the carpal and fetlock joints was evaluated by measuring the angular range of motion of these joints using a protractor and observing the knuckling gait and posture patterns of the animals as described by Govoni et al. [14], with minor modifications.

## Mild

Included in this category were patients who were able to stand on their own or with assistance, stand on the tip of the hoof even if their heels did not contact the ground, walk unsteadily and slowly, and whose foot or carpal joint position could be partially adjusted during the examination. The carpal lateral angle was measured to be between 75° and 65° in these patients.

## Moderate

Cases were classified as relating to this group if they needed assistance to stand, had a hoof tip that made contact with the ground at an angle, could not take more than a few steps before collapsing, and had an abnormal angle that couldn’t be corrected by passive flexion of the foot or carpal joints. It was found that the lateral angles of the affected carpal joints ranged from 65° to 35° in these patients.

## Severe

Included in this category, patients required assistance to stand but ultimately were unable to do because their feet touched the ground in a dorsal position, and their fetlock or carpal joints could not be brought back into alignment with passive movement. Carpal joint angulations were deemed less than 35° in these instances.

### Surgical reconstruction and postoperative management

The affected limb was surgically prepared with 70% alcohol and povidone-iodine (Povin^®^, Opsonin Pharmaceuticals Ltd., Bangladesh). The calves were positioned on lateral recumbency with the affected limb up. All calves were premedicated with atropine sulfate (Atrovet^®^, Techno Drugs Limited, Narsingdi, Bangladesh) at 0.04 mg/kg body weight (BW) intramuscularly and xylazine hydrochloride (Xyla^®^, Interchemie Werken, Holland) at 0.08 mg/kg BW intravenously. Linear infiltration analgesia on the incision site was performed with 2% Lidocaine Hydrochloride (Jasocaine Plus^®^, Techno Drugs Ltd., Narsingdi, Bangladesh).

**Figure 2.**
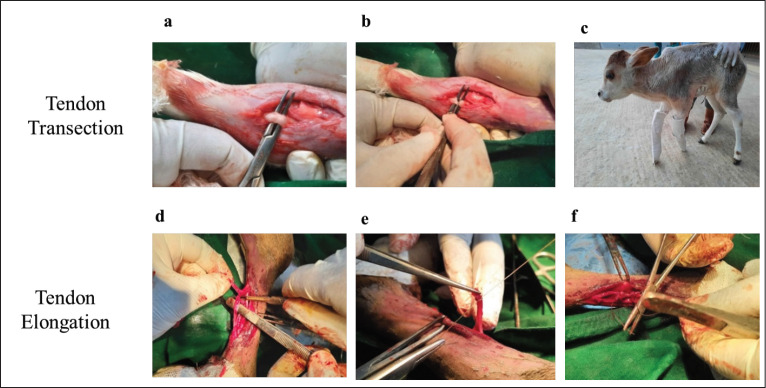
Surgical correction of knuckling (a) exteriorize of the superficial flexor tendon, (b) transecting the tendon, (c) full-limb cast fixation with PVC and POP, (d) Z-tenotomy (e) end-to-end suturing of the severed tendon using nylon, (f) muscle suture.

Depending on the degree of angulation and severity of the problem, the surgical procedure was performed in two ways: a) Tendon transection method and b) Tendon elongation method. To perform tendon transection on carpal knuckling, a longitudinal incision was created on the medial side of the leg parallel to the superficial digital flexor tendon (SDFT). After exposing the tendons, the transection was carried out sequentially on the SDFT and DDFT until the limb was sufficiently extended (Fig. 2a and b). In fetlock knuckling, tenotomies were performed on the medial and lateral sides of the joint after a superficial flexor tendon incision and firmly extending the fetlock joint. The skin was apposed with a sterile nylon suture. 

Regarding the tendon elongation method, the SDFT or DDFT was exposed, the capsule was incised, the tendon was cut in a Z-fashion, and an end-to-end tendon suture with sterile nylon suture was carried out (Fig. 2d and e). Synthetic absorbable suture (Vicryl^®^, Hican Angel Medical Instruments Co. Ltd., China) was used to unite the capsule and muscle (Fig. 2f). Simple interrupted nylon sutures were used to seal the skin. Postoperative external co-aptation of the limbs, the most important part of the limb straitening, was implemented with polyvinyl chloride (PVC) splints and Plaster of Paris (POP) cast (Fig. 2c). 

Postoperatively, the calves received antibiotic prophylaxis ceftriaxone (Trizon Vet^®^, Acme Laboratories Ltd., Dhaka, Bangladesh) at 30 mg/kg BW intramuscularly. In addition, chlorpheniramine maleate (Astavet^®^, Acme Laboratories Ltd., Dhaka, Bangladesh) at 1 mg/kg BW for 5 days and ketoprofen (Keto-A-Vet^®^, Acme Laboratories Ltd., Dhaka, Bangladesh) at 1 mg/kg BW of for 3 days were also administered. The animals’ owner was advised to restrict the mobility of the animals during the first week following surgery. A full-limb cast fixation with PVC and POP over the knuckled joint (s) was needed for more than 3 weeks’ post-surgery. The casts were removed when the calves were able to walk normally. Postoperative radiographs were taken on days 14 and 28 post-surgery to observe the straightening of the joint and to assess healing progress. 

### Postsurgical gait test

Following surgery, gait tests were performed on the operated calves on days 7 and 21. Based on the results of a gait test, calves were categorized as having lameness or not. Lameness with insufficient improvement in the fetlock and carpal knuckles was defined by incompetence to bear weight on the affected toe, lethargic movement, and a cranially tilting back line. Non-lameness was characterized as the capacity to put weight on the affected toe and to walk with a straight back after a satisfactory improvement in knuckling. All calves were evaluated for lameness 7 days after the operation. The PVC and POP were removed from non-lame calves 7 days after surgery, and no additional treatment was given. Whereas, for individuals that showed lameness, PVC and POP were applied to the affected limbs to enable them to walk again. At 21 days’ post-surgery, all calves undertook a gait test without external fixations. The prognosis was favorable for the calves who had successfully recovered from lameness. On the other hand, the prognosis was guarded to poor for the calves that showed lameness along with mild to moderate knuckling. 

### Biochemical examinations

Three ml of blood samples were drawn from the jugular vein of the knuckling patients on the day of the procedure prior to operation (D0) and on day 21 (D21) post-surgery. These whole blood samples were transferred to clot activator tubes and centrifuged at 3,000 rpm for 10 min to collect the serums and stored at −20°C for future use. Calcium, magnesium, vitamin D, iron, and zinc levels in serum samples were measured using T80 UV/VIS Spectrophotometer (USA) through the photometric method.

### Statistical analysis

All the data were expressed as Mean ± SEM (Standard Error of Mean). Statistical analyses were performed using a one-way analysis of variance factor-1 analysis with Statistical Package for the Social Sciences version 20.0. A probability *p* < 0.05 or less was considered statistically significant. 

## Results

### Prevalence and clinical features of flexor deformity

**Figure 3.**
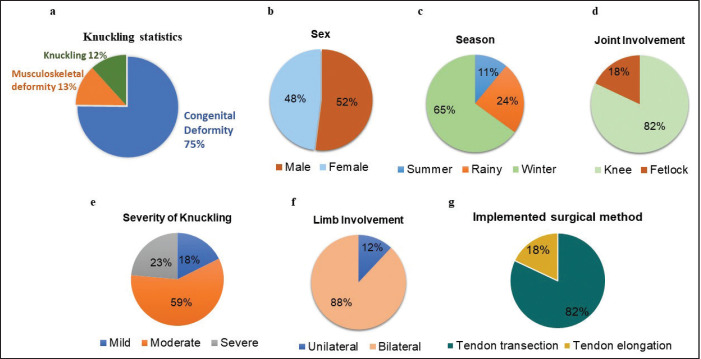
Epidemiology of knuckling. (a) prevalence of knuckling among the congenital deformity and musculoskeletal anomaly, distribution of knuckling based on (b) sex, (c) season, (d) joint involvement in knuckle calves, (e) severity of knuckling, (f) involvement of limb, i.e., unilateral or bilateral type, (g) surgical method employed to correct the knuckling.

Musculoskeletal system anomalies were recorded for 13% (*n* = 19) of the 109 newborn bovine calves admitted to BAU VTH with different congenital malformations, of which flexural deformity (knuckling) accounted for 12% of the total number of congenitally deformed cases (Fig. 3a). The knuckling patients presented to the hospital were between 1 and 6 days old. The male calves had a higher prevalence of knuckling (52%, *n =* 9) than those of the females (48%, *n = *8) (Fig. 3b). The knuckling calves were more frequently recorded during winter (65%, *n =* 11), followed by the rainy (24%, *n =* 4), and summer season had the lowest frequency (11%, *n =* 2) (Fig. 3c). The maximum calves with knuckling were recorded in the carpal joint (82%, *n =* 14). Only 3 (18%) patients had fetlock knuckling (Fig. 3d). The severity of knuckling was assessed by measuring the cranial angle of the carpal and fetlock joint as described in the materials and methods. In this study, 18% (*n =* 3) calves had mild type knuckling, 59% (*n =* 10) calves exhibited moderate type, and 23% (*n =* 4) had a severe type of knuckling (Fig. 3e). Clinical examination demonstrated that among 17 calves, only 2 (12%) had a unilateral deformity, while the majority of the calves (88%, *n =* 15) had bilateral knuckling (Fig. 3f). Of the 17 calves, 13 calves (76%) were treated by tendon transection techniques, whereas 4 calves (24%) were treated by tendon elongation procedures using Z-tenotomy method when the contraction of the tendon was excessive (Fig. 3g).

### Evaluation of surgical reconstruction of the flexural malformation

**Figure 4.**
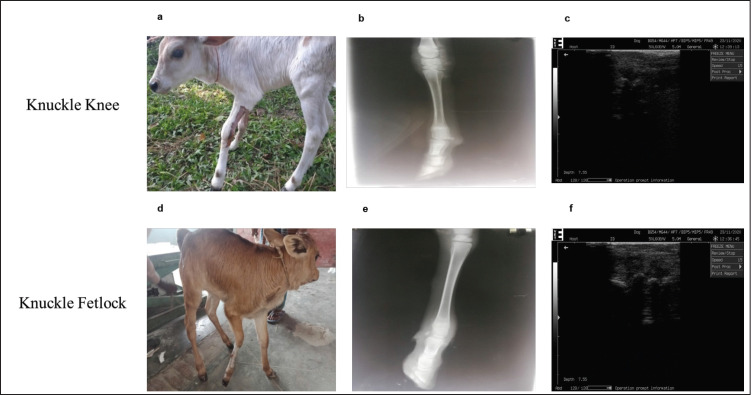
Postoperative clinical, radiographic, and ultrasonographic examination in calves a) clinical examination in knuckle knee b) radiographic examination in knuckle knee c) ultrasonographic examination in knuckle knee d) clinical examination in knuckle fetlock e) radiographic examination in knuckle fetlock f) ultrasonographic examination in knuckle fetlock.

All the patients were carefully monitored postoperatively to evaluate the prognosis. We have performed tendon transection in 13 calves, and 4 calves underwent for tendon elongation method. Based on the gait test performed on day 7 post-surgery after taking off the bandages, we observed that 9 out of 13 calves of the tendon transection method and 2 of 4 calves in the tendon elongation technique achieved non-lameness (Table 1). To avoid unwanted damage to the juvenile tendon and associated structure surrounding the joint that has been recently organized, the PVC splint bandages have been renewed and extended for another 2 weeks. At 21 days’ post-surgery, the remaining 5 calves in the tendon transection group and 1 of 2 lame calves of the tendon elongation group achieved non-lameness on the gait test (Table 1). The cumulative results indicate that 13 of 13 (100%) forelimbs with mild to moderate knuckling had a good prognosis with the tendon transection method. However, one calf of severe knuckling type did not respond to surgery, and the prognosis was bad over time; thus, 3 of 4 calves (75%) recovered after surgery with the tendon elongation method. Surgical complications such as edematous swelling and wound sepsis were observed in all the treated calves, which were successfully addressed. Postoperative radiography showed satisfactory straightening of carpal and fetlock deformity, and ultrasonography exhibited no abnormalities indicating that the ligament and surrounding structures have been reorganized (Fig. 4a–f). These findings suggested that the surgical treatment was effective in treating calves with mild to severe types of knuckling, allowing them to return to their normal activities.

**Table 1.** Postoperative gait test result in calves with knuckling.

Knuckling type	Operation technique		Gait test result on first-week post-surgery	Gait test result on 3-week post-surgery
	Tendon transection	Tendon elongation	(Non-lameness achievement)	(Non-lameness achievement)
Mild to moderate	13	0	69% (9/13)	100% (13/13)
Severe	4	4	50% (2/4)	75% (3/4)

### Biochemical findings

The serum calcium, magnesium, vitamin D, Zinc, and iron were evaluated in knuckling calves and were presented in Table 2. Postoperative follow-up values were compared with the respective patient’s preoperative values. In the animals of both knee and fetlock groups, the serum calcium level increased non-significantly (*p *> 0.05) on day 21. The serum magnesium level exhibited a significant (*p* < 0.05) increase on day 21 post-surgery. Vitamin D, which is thought to be an important factor in the development of flexural deformity, decreased significantly (*p* < 0.05) on day 21 postoperation in both types of knuckling, indicating that the excess of this vitamin may be associated with the genesis of congenital flexural deformity. Similar to vitamin D, the level of zinc and iron also decreased throughout the healing process and significantly lowered on day 21 post-operation in calves of both fetlock and carpal deformity. 

## Discussion

Congenital malformation of the locomotor system affecting the flexor and extensor tendons of the fetlock and pastern joints is prevalent in calves, lambs, and foals [15,16]. As a result, the animal cannot achieve or maintain the normal extension of the limbs. The forelimbs are more frequently affected by this ailment, though it can affect the flexor tendon of either or both hind limbs. The majority of the knuckling observed within the first few days of birth, as reported by Shivaprakash and Kumar [17] and Niwas et al. [18], which is similar to our finding where the young claves of 1 to 6 days’ old had knuckling problem.

In this study, the number of male calves with flexural deformity was higher than the females, similar to previous researchers [7,8]. Because male calves gain more weight at birth than female calves, there may be a weight disparity between the fetus and the mother, limiting the fetus’s usual mobility within the uterus and eventually leading to the development of constricted limbs. We observed bilateral flexural deformity in all the calves, similar to the findings of Sato et al. [19] and Baltaci et al. [9], who reported a greater bilateral flexural deformity in bovine calves than unilateral deformity. We have recorded a higher prevalence of fetlock deformity than knee or carpal type. This was dissimilar to the previous report of Ochube et al. [20], who stated that the knee joint was more prone to develop deformity than the fetlock joint. Fazili et al. [8] reported forelimb knuckling at a higher prevalence, similar to our finding. We have recorded all the cases in the front leg. However, it may be postulated that vascular contraction is a regular phenomenon for thermoregulation in animals during winter. Due to vascular contraction, the blood circulation to the fetal limb may be restricted, which could cause abnormal joint structure and flexural deformity.

**Table 2.** Biochemical changes in calves with knee and fetlock knuckling.

Parameter	Knuckling type	Day 0	Day 21
Calcium	Knee	12.034 ± 0.50^a^	13.026 ± 0.80^a^
	Fetlock	9.267 ± 0.20^a^	9.713 ± 0.743^a^
Magnesium	Knee	1.923 ± 0.005^a^	1.943 ± 0.005^b^
	Fetlock	1.88 ± 0.01^a^	1.83 ± 0.05^b^
Vitamin -D	Knee	101.400 ± 0.80^a^	31.300 ± 0.20^b^
	Fetlock	92.46 ± 0.846^a^	32.24 ± 4.61^b^
Zinc	Knee	115.00 ± 2.00^a^	105.00 ± 2.00^b^
	Fetlock	126.00 ± 2.00^a^	100.00 ± 2.00^b^
Iron	Knee	515.00 ± 2.00^a^	501.00 ± 2.00^b^
	Fetlock	517.00 ± 2.00^a^	507.00 ± 2.00^b^

Data were presented as mean ± SEM values; Values with a different superscript letter (a, b) in the same raw differed significantly at *p* < 0.05.

Based on the clinical characteristics and the angulation of the deformed extremity, some researchers divided flexural deformity into three categories: mild, moderate, and severe, but this classification, arthrometric, or goniometric data were not taken into consideration [8]. A straightforward, affordable, trustworthy, impartial, and non-invasive technique for deformed joint classification is goniometry Govoni et al. [14] which has been followed in this study. The carpal deflection angle in the study ranged from 35° to 65°. In a related study on fetlock deformity, Fazili et al. [7] discovered that the deformity ranged from 30° to 65° based on goniometry.

According to Simon et al. [15] and Anderson et al. [2], treatment of flexural deformity should be initiated immediately after recognition of the problem. Although the very mild form may be treated non-surgically [21,7], surgery is the definitive treatment for most categories. In this study, two techniques, namely the tendon transection method and tendon elongation method, have been employed to correct the deformity based on the type and severity of knuckling, with a success rate of 92% and 100%, respectively. Rashmi et al. [22]; Sato et al. [19] performed transection technique on the SDFT and DDFT in calves with severe MPFD. They have researched 17 limbs of 10 calves with a success rate of 84%. We never dissected the DDFT; instead, we transected the SDFT and stitched the DDFT from end to end before externally fixing it and casting the entire limb. This may be the reason behind our better result. Factors including improper uterine posture, a large calf on the heifer, inadequate nutrition, goiter, viral infections, neuromuscular problems, elastin and collagen defects, and mineral imbalance are said to have a significant part in the development of congenital tendon diseases and disorders [7]. Compared to preoperative and postoperative values, we have seen a significant change in magnesium, vitamin D, zinc, and iron in the affected calves in this study. The serum levels of zinc and vitamin D were found to be significantly lower on day 21 post-surgery than before surgery, indicating that the excess or imbalance of these trace elements and vitamin D may be closely associated with the development of flexural deformity. Genccelep et al. [8]; Galloway et al. [23] stated the values of zinc and vitamin D were markedly lower in postoperative conditions as compared with the control group, which is similar to our findings. Myotonic dystrophy type II is correlated with zinc imbalance which may contribute to developing flexural deformity in calves [9, 24]. 

However, it is important to ascertain the status of the following surgery’s tendons, ligaments, and associated structures to evaluate the soundness of these structures postoperatively. For this reason, ultrasonographic examination at regular intervals for a couple of months should be performed, which was lacking in this study.

## Conclusion

We conclude that forelimb bilateral knuckling is one of the most prevalent congenital musculoskeletal deformities affecting newborn bovine calves. Iron, zinc, and vitamin D imbalance are closely associated with the development of flexural deformity, which needs to be addressed in the pregnant dam. Neonatal calves with fetlock or carpal knuckling are satisfactorily treatable by SDFT transection and tendon elongation by Z-tenotomy. Nevertheless, early detection and the commencement of treatment are necessary to improve the outcome. 

## List of Abbreviations

BW, Body Weight.
